# Optimal designs for population pharmacokinetic studies of oral artesunate in patients with uncomplicated falciparum malaria

**DOI:** 10.1186/1475-2875-10-181

**Published:** 2011-07-01

**Authors:** Kris M Jamsen, Stephen B Duffull, Joel Tarning, Niklas Lindegardh, Nicholas J White, Julie A Simpson

**Affiliations:** 1 Centre for Molecular, Environmental, Genetic and Analytic Epidemiology, School of Population Health, The University of Melbourne, Melbourne, Australia; 2 School of Pharmacy, The University of Otago, Dunedin, New Zealand; 3 Mahidol-Oxford Tropical Medicine Research Unit, Mahidol University, Bangkok, Thailand; 4 Centre for Tropical Medicine, Churchill Hospital, Oxford, UK

## Abstract

**Background:**

Currently, population pharmacokinetic (PK) studies of anti-malarial drugs are designed primarily by the logistical and ethical constraints of taking blood samples from patients, and the statistical models that are fitted to the data are not formally considered. This could lead to imprecise estimates of the target PK parameters, and/or designs insufficient to estimate all of the parameters. Optimal design methodology has been developed to determine blood sampling schedules that will yield precise parameter estimates within the practical constraints of sampling the study populations. In this work optimal design methods were used to determine sampling designs for typical future population PK studies of dihydroartemisinin, the principal biologically active metabolite of oral artesunate.

**Methods:**

Optimal designs were derived using freely available software and were based on appropriate structural PK models from an analysis of data or the literature and key sampling constraints identified in a questionnaire sent to active malaria researchers (3-4 samples per patient, at least 15 minutes between samples). The derived optimal designs were then evaluated via simulation-estimation.

**Results:**

The derived optimal sampling windows were 17 to 29 minutes, 30 to 57 minutes, 2.5 to 3.7 hours and 5.8 to 6.6 hours for non-pregnant adults; 16 to 29 minutes, 31 minutes to 1 hour, 2.0 to 3.4 hours and 5.5 to 6.6 hours for designs with non-pregnant adults and children and 35 to 59 minutes, 1.2 to 3.4 hours, 3.4 to 4.9 hours and 6.0 to 8.0 hours for pregnant women. The optimal designs resulted in acceptable precision of the PK parameters.

**Conclusions:**

The proposed sampling designs in this paper are robust and efficient and should be considered in future PK studies of oral artesunate where only three or four blood samples can be collected.

## Background

The World Health Organization recommends artemisinin-based combination therapy (ACT) as first line treatment for uncomplicated *falciparum *malaria in all malaria endemic areas [1]. ACT involves treatment with two or more different anti-malarial drugs - a highly effective but short-lived artemisinin derivative, and a less potent but longer-lived partner drug(s). Most anti-malarial drugs were introduced with incomplete dose-finding studies, so the dosage, particularly in some important patient groups is too low. This is especially true for young children and pregnant women, who carry much of the malaria burden and are at greatest risk of treatment failure [2].

To treat malaria effectively, the dose and frequency of administration of the anti-malarial drug need to provide drug concentrations over time sufficient to kill all of the parasites in the body. This drug concentration-time profile is determined by the pharmacokinetic (PK) properties of the drug [3]. Previous studies have shown that anti-malarial pharmacokinetics for children and pregnant women can differ substantially from non-pregnant adults given the same dosing regimen [4,5]. Therefore, there is an urgent need to characterise the concentration-time profile in vulnerable patient groups so that optimal dosing regimens can be determined.

For young children and pregnant women with uncomplicated malaria the invasiveness of taking many blood samples is not acceptable to the patient or parent. Thus the intensive blood sampling that would make for ideal population PK modeling is not feasible, so the times at which the blood samples are taken must be chosen carefully so that information is maximised and the PK parameters can be estimated with reasonable precision. This requires knowledge of the structural PK model for the drug(s) being studied and formal consideration of how the population PK model is fitted to the data. Given that there is such limited opportunity to take blood samples, failing to consider the statistical techniques required to estimate the target parameters can lead to designs that are inefficient (provide imprecise estimates of model parameters) and in some cases study failure. This wastes time and money and is arguably unethical since volunteers participating in these studies have contributed their time, discomfort and biological samples needlessly. Hence there is a scientific and ethical incentive to design population pharmacokinetic studies so that precise parameter estimation is achievable [3].

The theory of optimal design has been developed to propose the ideal timing of blood sampling when designing population PK studies [6]. Optimal design methods incorporate knowledge of the PK profile of the drug, the practical constraints of blood sampling and the statistical techniques used to estimate the PK parameters. The resulting optimal design yields the lowest possible values of the standard errors of the estimated PK parameters from within the practical constraints of sampling patients. To date, this methodology has not been applied to population PK studies of anti-malarial drugs. Previous works in other areas have concentrated on providing a case specific design (e.g. Waterhouse *et al*. [7]), whereas the intention of this work is to provide designs that can be applied to a wide range of future studies.

This investigation focuses on dihydroartemisinin (DHA) following oral administration of artesunate (AS), the main artemisinin derivative used to treat uncomplicated *falciparum *malaria. AS is rapidly hydrolysed to DHA, its principal biologically active metabolite. Most of the anti-malarial activity of artesunate is explained by DHA. These class of drugs reduce the number of parasites in the body faster than any other anti-malarial drugs [8]. Oral AS is rapidly absorbed and eliminated from the body, providing about 6-8 hours to take blood samples post-dosing.

The overall aim of this work was to provide a set of designs that can be used prospectively to study DHA following oral AS administration for all target populations, which include non-pregnant adults, children and pregnant women. This was achieved by:

(i) determining appropriate structural PK models and parameter values for DHA for the target populations,

(ii) identifying the practical constraints of taking blood samples from patients,

(iii) determining optimal designs using the information above, and

(iv) evaluating the optimal designs.

## Methods

### Overview of optimal design

In brief, an optimal design for a population PK model (i.e. a nonlinear mixed-effects model) is the design (i.e. set of blood sampling times) out of all possible designs that will maximise the determinant of the population Fisher information matrix, thereby minimising the standard errors (i.e. maximising the precision) of the parameter estimates. This is more specifically a D-optimal design, but will be referred to as an optimal design throughout this paper. The method for computing the population Fisher information matrix using a first-order approximation of a nonlinear mixed-effects model is given elsewhere [6,9]. Software has been developed to determine optimal designs for nonlinear mixed-effects models [10], where the user specifies the structural PK model, the values of the PK parameters, the between-subject variability of the PK parameters, the residual variability and an initial sampling scheme. Several models can be entered simultaneously (competing models), and restrictions on potential sampling times can be made to ensure that the final optimal sampling times are within the constraints of the study. After this information is entered, an optimisation algorithm is used to search for the optimal design, where the design space (i.e. the set of all possible designs) is limited to the specified restrictions on the sampling schedule. The output includes the optimal sampling times and the expected standard errors of the model parameter estimates assuming the optimal design. For technical details see [9].

### Determining pharmacokinetic models and parameter values for the designs

Structural PK models and PK parameter values for DHA in non-pregnant adults were determined by analysing a subset of DHA PK data with intensive blood sampling [11]. The data were derived from studies of non-pregnant adult patients with uncomplicated *falciparum *malaria attending field clinics on the Thai-Burmese border (Wang Pha) and western Cambodia (Pailin) who were given 4 mg/kg of oral AS. Six to twelve venous blood samples were taken from 32 patients over the first 12 hours post-dosing and assayed to determine DHA concentrations. Plasma concentrations of AS and DHA were measured by means of high-throughput liquid chromatography-tandem mass spectrometry after solid phase extraction [12]. The coefficient of variation for all quality control samples was less than 5% at all drug concentrations. Full details of the study have been published [11]. For all PK models, it was assumed that AS was completely metabolised into DHA. This assumption does not affect the relevance and predictive performance of the model since only DHA was of interest. The statistical analysis was performed in NONMEM version VI [13] in conjunction with Wings for NONMEM[14]. In NONMEM the FOCE (first-order conditional estimation) with INTERACTION method was used.

The results from the analysis of the non-pregnant adult data were used to define oral clearance and apparent volume of distribution parameters for children and infants via allometric scaling, which scales these parameters by individual body weight relative to a reference body weight. In addition, maturation on oral clearance was also incorporated. These calculations were performed since there are few published PK studies of oral AS in children with intensive blood sampling and very limited data published on anti-malarial drug concentrations in infants [15]. For technical details of these calculations see Additional file 1.

The structural PK models and values of their parameters for pregnant women were obtained from McGready *et al*. [5], which is the only published population PK study of oral AS in pregnant women with presenting *falciparum *malaria.

### Identifying sampling constraints

To identify the practical constraints of obtaining blood samples from malaria patients, a survey was e-mailed to 22 malaria researchers (including clinicians, pharmacologists and health workers) with extensive experience in conducting anti-malarial PK studies in Asia and Africa. JAS wrote the survey, and it was reviewed by NJW and Prof Richard Price (Menzies School of Health Research, Darwin, Australia). Information was collected on patient and logistical factors that restrict the number of blood samples that can be taken from an individual, possible times samples could be taken, feasible methods of taking blood, how many samples could be taken from individuals within 8 and 24 hours post dosing, the maximum number of samples that could be taken from an individual and the minimum time between consecutive samples. The latter four items were noted for adults, children and pregnant women by field setting (hospital or community patients).

### Determination of the optimal designs

Optimal designs for future population PK studies of DHA in adults, children and pregnant women were determined using POPT[16]. The designs were based on the determined structural PK models, parameter values, likely values of covariates (i.e. age and/or weight) and key constraints identified in the questionnaire.

For all designs, competing models were entered into POPT to incorporate uncertainty in the structural PK models and/or parameter values. For the structural PK models, this was done by defining each plausible model in POPT. Then for each structural PK model defined, the PK parameter values were set to the population mean estimates and the lower and upper bounds of their respective 95% confidence intervals (thus three sets of PK parameter values were considered for each structural model). Furthermore, 95% weight was allocated to the models with the PK parameters set to the population estimates. The designs were then determined by optimising over all competing models simultaneously. This method for robust design has been used previously [17] and has been shown to be as effective as ED designs but without the computational burden [18].

To provide flexibility for taking blood samples in the field, sampling windows were computed in POPT for each optimal sampling time. A sampling window is a time interval containing the optimal sampling time, where any blood sample taken within the interval ensures minimal impact on the precision of the estimated parameters. POPT determines the windows via a three-stage sampling procedure where samples are generated randomly and those that achieve the necessary level of efficiency are accepted. For further details see [16]. For the determined designs, the windows were constructed such that there would be a minimal increase in the standard errors as defined by a 20% reduction in the overall statistical efficiency relative to the optimal sampling times.

### Evaluation of the optimal designs

The robustness of the optimal designs was evaluated by a simulation-estimation procedure that was automated in NONMEM. So for each optimal design:

1. 100 datasets were simulated, where each dataset consisted of virtual individuals that were randomly selected from a hypothetical population that matched the characteristics (i.e. ages and/or weights) of the target population (e.g. non-pregnant adults, children or pregnant women).

2. Individual DHA concentrations were simulated at the optimal sampling times from a respective population PK model.

3. Each simulated dataset was analysed with NONMEM using the FOCE with INTERACTION method.

4. For each estimated parameter, the standard deviation of the estimates from the 100 analyses was computed. These standard deviations of the estimated parameters can be regarded as empirical standard errors. The empirical standard errors were then expressed as percent relative standard errors (%RSEs), where the %RSE is given by 100 multiplied by the empirical standard error divided by the median parameter estimate from the 100 analyses. The empirical %RSEs were compared with the expected %RSEs from POPT.

For the simulation of the virtual individuals (step 1), non-pregnant adult weights were simulated based on the non-pregnant adult PK DHA data from the Thai-Burmese border and Western Cambodia [11]. For designs with children, ages and weights were simulated from a linear model based on data from Simpson *et al*. [19]. Weights for pregnant women were derived from the weight distribution reported in McGready *et al*. [5].

## Results

### Pharmacokinetic models and parameter values for the designs

**Figure 1:**
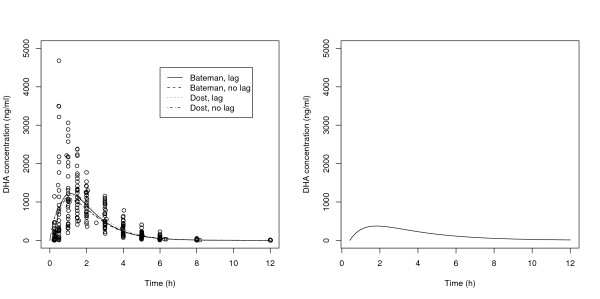
**Population pharmacokinetic profiles of DHA for pregnant and non-pregnant adults**. The left graph displays the DHA concentrations from the 32 non-pregnant adults reported in [11] and the population pharmacokinetic profiles that were estimated from this data. The right graph shows the population pharmacokinetic profile for pregnant women reported in [5].

The left graph of Figure 1 displays the DHA PK data reported in non-pregnant adult patients (n = 32) [11]. Table 1 displays the results from analysing this data and hence the PK models and parameter values that were used for determining the optimal designs for non-pregnant adults. From the analysis, four (competing) structural PK models were selected for determining optimal designs for non-pregnant adults: Bateman without a lag-time, Bateman with a lag-time, Dost without a lag-time and Dost with a lag-time. The mathematical details of these models are given in Additional file 1.

**Table 1:** Population pharmacokinetic models and parameter values for the non-pregnant adults and pregnant women designs

	Non-pregnant adults		Pregnant women^♭,♮^
		
Model &Parameter*^,§^	Estimate^† ^(95% CI)^◇^	Estimate^‡ ^(95% CI)^◇^	Estimate^† ^(95% CI)^◇^
**Bateman model**			
*k_a _*(/h)	0.82 (0.76, 0.87)	0.89 (0.81, 0.97)	1.19 (0.78, 1.60)
*CL*/*F *(L/h)	47.5 (44.6, 50.4)	48.8 (42.3, 55.3)	88.5 (60, 117)
*V*/*F *(L)	32.1 (24.6, 39.6)	44.4 (30.2, 58.6)	232 (57.0, 406)
*t_lag _*(h)	0.21 (fixed)	n/a	0.42 (0.34, 0.50)
	26.7	11.4	not reported
Ω*_CL_*_/_*_F_*	29.4	26.8	47.0
Ω*_V_*_/_*_F_*	81.9	64.7	154
	10.0	n/a	not reported
Ω*_CL_*_/_*_F_*,*_V_*_/_*_F_*	0.58	0.75	not reported
	0.19	n/a	not reported
*σ*	0.41	0.55	not reported

**Dost model**			
*k *(/h)	0.99 (0.91, 1.1)	0.95 (0.86, 1.03)	
*V*/*F *(L)	46.3 (37.6, 55.0)	51.3 (41.2, 61.4)	
*t_lag _*(h)	0.21 (fixed)	n/a	
Ω*_k_*	22.0	20.8	
Ω*_V_*/*_F_*	48.2	42.2	
	11.4	n/a	
Ω*_k_*_,_*_V_*/*_F_*	-0.79	-0.81	
*σ*	0.47	0.57	

*The Ω's represent between-subject variances and covariances, expressed as the percent coefficient of variation (%CV, approximated by the square root of the variance estimate multiplied by 100) and correlation coefficients, respectively

^§^The *σ*'s represent residual standard deviations (proportional)

^♭^Taken from McGready *et al*. [5]

^♮^Reported *CL*/*F *(L/kg/h) and *V*/*F *(L/kg) multiplied by median weight (50 kg)

^†^Lag-time included

^‡^Lag-time not included

^◇^Upper and lower bounds of CIs were considered for the PK parameters only

Table 1 also displays the parameter values of the Bateman model for pregnant women proposed by McGready *et al*. [5], and the right graph of Figure 1 shows the corresponding population PK profile. The authors did not estimate the between-subject variability (BSV) of the absorption rate constant  and the residual standard deviation (*σ*) was not reported, so the values of these parameters were set to those of the non-pregnant adults for the determination of the optimal designs.

PK parameters for children were based on results from the Bateman models for the non-pregnant adults in Table 1 and applying maturation to *CL*/*F *and allometric scaling to *CL*/*F *and *V*/*F*. The Dost model was not considered since it is a simpler version of the Bateman model where only *V*/*F *can be scaled. The age/weight combinations required for the scaling and maturation were based on a linear model using data from [19] (more details of the age/weight combinations for the designs are given below and in Additional file 1, Figure S2).

### Sampling constraints

Sixteen of the 22 malaria researchers contacted (73%) responded to the questionnaire regarding blood sampling of malaria patients. The reported number of blood samples that could be taken from patients in community settings within 8 hours post dosing ranged from one to six for adults (pregnant and non-pregnant) and one to four for children. For all patient groups, responses for the minimum time between blood samples (for any anti-malarial drug) ranged from 15 minutes to 24 hours.

Given these constraints, it was decided that adults should have four blood samples taken whereas children should have three, where consecutive samples from an individual patient would be at least 15 minutes apart. Three to four samples per patient would allow a single sampling schedule (at least for the adults), which is appealing for clinical implementation, and would require only a moderate number of patients (i.e. 50-100) to achieve acceptable parameter precision. It was also felt that a minimum of three samples per patient would provide means for distinguishing between residual and between-subject variability, which have been reported to be large for DHA [19].

### Optimal designs

Using the PK models and parameter values given in Table 1 and the sampling constraints described above, three sets of designs were determined in POPT:

(i) non-pregnant adults only,

(ii) non-pregnant adults and children, and

(iii) pregnant women only.

Separate designs for the non-pregnant adults and pregnant women were considered reasonable since adequate sampling could be done in both groups and there was evidence for substantial differences in their population PK profiles. Children were included in a design with adults since only minimal sampling could be performed in children, and consisted of four age groups: <2 years, 2-10 years, 11-20 years and >20 years (the age groups were determined from the questionnaire).

All designs consisted of 60 individuals, which was considered logistically feasible and a necessary minimum for parameter estimation. For all designs it was specified in POPT that the optimal sampling times had to be at least 15 minutes apart over a dosing interval of 24 hours. Table 2 displays the optimal sampling times and sampling windows from POPT for each design. A graphical display of the optimal times and windows is provided in Additional file 1, Figure S1.

**Table 2:** Optimal sampling times and sampling windows from POPT for each design

Design	Optimal times (sampling windows)			
Non-pregnant adults* (n = 60)	20 min (17 to 29 min)	35 min (30 to 57 min)	3.0 h (2.5 to 3.7 h)	6.2 h (5.8 to 6.6 h)
Non-pregnant adults and children* <2 y (n = 10):	19 min (16 to 28 min)	2.3 h (2.0 to 2.8 h)	5.8 h (5.5 to 6.1 h)	
2-10 y (n = 10):	19 min (16 to 26 min)	34 min (31 min to 1.0 h)	5.76 h (5.5 to 6.2 h)	
11-20 y (n = 10):	20 min (17 to 29 min)	2.2 h (2.1 to 3.0 h)	6.1 h (5.6 to 6.5 h)	
>20 y (n = 30):	20 min (18 to 27 min)	2.5 h (2.2 to 2.7 h)	2.8 h (2.7 to 3.4 h)	6.1 h (5.9 to 6.6 h)
Pregnant women*^,†,‡^ (n = 60)	43 min (35 to 59 min)	3.3 h (1.2 to 3.4 h)	3.5 h (3.4 to 4.9 h)	7.4 h (6.0 to 8.0 h)

*For structural models with a lag-time, the lag-time parameter and its BSV were specified in POPT to not be estimated

^†^The BSV of *k_a _*was set to that of the non-pregnant adults and the BSV of *V*/*F *was reduced to 1

^‡^Competing values of the BSV of *V*/*F *were set to 0.67 (derived from the results of the non-compartmental analysis in [5]) and 2.37 (the reported BSV from the population PK analysis in [5])

### Evaluation of the optimal designs

Tables 3 and 4 display the results from the simulation-estimation procedure using the Bateman and Dost models for analysis (respectively) along with the expected %RSEs of the model parameters from POPT assuming the optimal sampling times. For all designs, data were simulated from and analysed with the Bateman model with a lag-time. This was done since this model provided the best fit to the non-pregnant adult data and was reported in McGready *et al*. [5]. In addition, the Dost model was used to analyse the simulated data from the non-pregnant adult design. For the simulation step of the non-pregnant adult only and non-pregnant adult and children designs, parameter values were set to the population estimates from the non-pregnant adult analysis (allometric scaling and maturation was used for the latter design). For the simulation step of the pregnant women designs, the parameter values were set to those identified in McGready *et al*. [5] with the BSV of *k_a _*set to that of the non-pregnant adults and the BSV of *V*/*F *reduced to 1 (see Tables 3 and 4 for details).

**Table 3:** Expected and empirical percent relative standard errors (%RSEs) for the Bateman model assuming the optimal designs

	*k_a_*	*CL*/*F*	*V*/*F*	*t_lag_*		Ω*_CL_*_/_*_F_*	Ω*_V_*_/_*_F_*	*σ*
**Non-pregnant adults**								
Optimal design								
POPT*	5.44	3.73	10.15	-	45.5	22.2	17.5	4.98
Simulation-estimation^†,‡^	10.7	5.99	17.3	6.86	52.9	30.7	21.6	7.87

**Non-pregnant adults and children**								
Optimal design								
POPT*	5.14	3.81	10.4	-	42.5	23.6	19.0	5.83
Simulation-estimation^†,‡^	8.97	5.63	22.7	9.88	55.2	37.4	29.2	8.52

**Pregnant women**								
Optimal design								
POPT*	8.22	4.76	10.5	-	-	15.9	14.7	4.20
Simulation-estimation^†,#^	14.7	8.28	17.5	8.67	-	27.0	25.8	7.33

*Expected %RSEs

^†^Empirical %RSEs

^‡^ fixed

^#^ and  fixed

**Table 4:** Expected and empirical percent relative standard errors (%RSEs) for the Dost model assuming the optimal designs

	*k*	*V*/*F*	*t_lag_*	Ω*_k_*	Ω*_V_*_/_*_F_*	*σ*
**Non-pregnant adults**						
Optimal design						
POPT*	2.32	5.06	-	16.7	17.3	4.31
Simulation-estimation ^†^^‡^	4.33	8.05	8.77	24.7	19.4	7.46

*Expected %RSEs

^†^Empirical %RSEs

^‡^ fixed

For all designs it was initially attempted to estimate all parameters, but this resulted in NONMEM being unsuccessful with minimising the objective function for reasons other than rounding errors in many of the runs. To improve this, the BSV of *t_lag _*was fixed to the value it was simulated at for all designs, and for the pregnant women design the BSV of *k_a _*was also fixed to the value it was simulated at. After these modifications, all models from the non-pregnant adult only and non-pregnant adult and children designs converged, and 96 of the models converged for the pregnant women design. The empirical %RSEs from all designs were consistently higher than the expected %RSEs from POPT, but still acceptable (<25% for the PK parameters and ≤55% for the BSVs). In addition, for the non-pregnant adults and children design, the maturation of oral clearance assumption was assessed by performing two additional simulation-estimations, varying the value of *k_mat _*(see Additional file 1 for an explanation of this parameter). Both of these simulation-estimations yielded similar results to those presented in Table 3.

To highlight how substantial the decrease in parameter precision can be by taking very few samples per patient, naïve designs, that is less intensive sampling schedules based on sampling constraints consistent with responses from the questionnaire and passing consideration of PK profiles, were evaluated via the simulation-estimation process described in the Methods section for all patient populations. See Additional file 1 for details of the naïve designs and the results from the evaluation (Additional file 1, Table S1).

## Discussion

This work determined optimal study designs for future population PK studies of DHA following oral AS for all key target populations where only a small number of blood samples can be taken. The proposed sampling schedules resulted in acceptable precision of the PK and between-subject variance parameters. 

The optimal designs were determined using population PK models from Asian populations, however the resulting sampling schedules should also be applicable to African populations. Models from Asian populations were used since knowledge of the population pharmacokinetics of DHA following oral AS in African patients with presenting *falciparum *malaria is scant. Stepniewska *et al*. performed a population PK study of AS and DHA in African children with uncomplicated malaria [20], however the data were very sparse (one sample per child for a given dose) and between-subject variability was reported for only one PK parameter (*CL*/*F*). A very recent study reported the population pharmacokinetics of DHA following oral AS in pregnant and non-pregnant women in Africa [21], but these women were asymptomatic and displayed low-grade parasitaemia. Also, the authors noted that their results were comparable to McGready *et al*. [21]. Therefore it was decided to not use the results from [20] or [21] and instead base the designs on an analysis of the rich non-pregnant adult data from [11] and the reported model for pregnant women in [5]. Though Asian and African populations may differ in factors that affect the PK (e.g. in terms of weight and Cytochrome P450 enzyme polymorphism), uncertainty was incorporated into the PK parameters to ensure the designs were robust to some alternative population PK profiles. To check this, the optimal designs were evaluated *post hoc *in POPT assuming PK profiles from individuals weighing 70 kg (via allometric scaling of the Asian *CL*/*F *and *V*/*F *parameters) and yielded similar expected precision to that shown in the Results section. Furthermore, results from [21] and published non-compartmental analyses of DHA following oral AS in African populations [22-24] were examined. The displayed data from [22] appeared to closely follow the population PK profile for children used for the designs, however [21], [23] and [24] showed somewhat different profiles than those used for the non-pregnant adults and pregnant women designs. Using the displayed data in [23] and [24], Bateman model parameters were approximated via nonlinear regression, and a Bateman model with zero-order absorption was approximated from the results in [21]. The optimal designs were again evaluated *post hoc *in POPT assuming these approximated profiles (the BSVs from the Asian populations were used for models approximated from [23] and [24]). For the models approximated from [23] and [24], the optimal designs gave acceptable precision for *CL*/*F *and *V*/*F *(and their BSVs), but required *k_a _*and/or its BSV to be fixed. In the case of the approximated zero-order absorption model, the BSV of *V*/*F *was required to be fixed for the pregnant women design. Therefore when studying African populations it is recommended to vary sampling within the windows, especially within the absorption phase, to gain more knowledge of the population PK profiles for this group. In particular, it is recommended to take some samples later in the windows, as results from [23,24] and [21] suggest that Africans may display a longer time to maximum concentration. As more population PK studies are performed in Africans, the optimal designs can be updated to cater for any observed differences. Additionally, it is also recommended to vary sampling within the windows for Asian populations, as this may provide more information about other plausible structural PK models (e.g. a two-compartment model), increased flexibility for parameter estimation and a safeguard against the possibility that the structural models and parameter values the designs were based on were incorrect.

The pregnant women design was based on models obtained from McGready *et al*. [5], which was the only published work found on the population pharmacokinetics of DHA in pregnant women with presenting *falciparum *malaria. The BSV for *V*/*F *was extremely large, which is likely due to *V*/*F *soaking up the between-subject variability of *k_a_*, which was not estimated. To account for at least some between-subject variability of *k_a _*in the design, the BSV of *k_a _*was set to that of the non-pregnant adults. To compensate for the inclusion of the BSV for *k_a_*, the population value for the BSV of *V*/*F *was reduced. It is therefore possible that alternative sets of PK parameter values exist for this group, so as previously noted sampling should be varied within the windows and as more PK data become available from pregnant women the optimal design will be re-evaluated. In addition, if the investigator expects to use an analytical method for measuring DHA concentrations that is less sensitive than that used in [11], it is recommended to take the last blood sample in pregnant women earlier within the sampling window to ensure that the concentration is above the lower limit of quantification. This should also be done for children if a less sensitive analytical method is used.

For all designs, the empirical %RSEs were consistently higher than the expected %RSEs from POPT. This observed difference may have been due to the way covariates (i.e. weight and age) were dealt with in POPT and the simulation-estimation procedure. In POPT, likely values for age and weight were assumed (i.e. weight for the adult designs and a range of age/weight combinations for each age group of the design with children). However, for the simulation-estimations, distributions were assumed for age and weight based on these likely values. Though this difference in procedures may have contributed to the consistently higher empirical %RSEs, the empirical %RSEs were still acceptable for both the PK parameters (<25%) and BSVs (≤55%). Furthermore, the %RSEs from the population Fisher Information matrix are lower bounds, so it is not surprising that the empirical %RSEs were larger. Therefore the derived optimal designs should be robust to plausible values of age and weight, and as more information is collected on the effect of these covariates on the PK parameters the designs can be re-evaluated.

The optimal sampling schedules proposed in this work were based on the key constraints of three to four blood samples per patient, a minimum of 15 minutes between consecutive samples and a sample size of 60 individuals. Thus they are the optimal sampling times assuming these key constraints. However, it is recommended to take additional blood samples, if at all possible, and if convenient within the sampling windows. Additional samples will provide even more information for the estimation of the model parameters and may assist with the exploration of other plausible structural PK models and/or parameter values.

## Conclusions

The use of optimal design methodology provided an analytical yet flexible framework to determine robust and efficient designs for future population PK studies of DHA following oral AS for all target populations, including pregnant women and children.

Current dosing recommendations for oral AS provide a resistance selection opportunity in patients with low drug concentrations and high parasite burdens, which are often children and pregnant women [25]. More population PK studies that are optimally designed, and using pharmacokinetic-pharmacodynamic (PK-PD) models are required to determine dosing regimens that achieve effective drug concentrations in all malaria patients [26,27]. The optimal designs proposed in this paper can be used in future studies to obtain precise PK parameter estimates under heavy sampling constraints, and can be considered a prototype for an iterative open access design support tool to help investigators studying anti-malarial efficacy and pharmacology in field studies. These will be provided by the Clinical Pharmacology module of the Worldwide Antimalarial Resistance Network [26].

## List of abbreviations

KMJ: Kris M Jamsen; SBD: Stephen B Duffull; JT: Joel Tarning; NL: Niklas Lindegardh; NJW: Nicholas J White and JAS: Julie A Simpson.

## Competing interests

The authors declare that they have no competing interests.

## Authors' contributions

JAS and KMJ conceived the project. JAS and NJW conducted the survey and KMJ, JAS and SBD implemented the models in NONMEM and the designs in POPT. KMJ wrote the first draft of the manuscript. SBD, JT, NL, NJW and JAS revised the manuscript critically for important intellectual content. All authors read and approved the final manuscript.

## Supplementary Material

Additional file 1 **This file explains the mathematical details of applying allometric scaling and maturation to PK parameters, the Bateman model and the Dost model**. The results from the evaluation of the naïve designs are also displayed [28-31]. Click here for file
