# Supplementary material for: Optimal designs for population pharmacokinetic studies of oral artesunate in patients with uncomplicated falciparum malaria
Source: Malar J. 2011 Jul 1;10:181. doi: 10.1186/1475-2875-10-181 (PMC3155838; doi:10.1186/1475-2875-10-181)
Supplement: Additional file 1 — This file explains the mathematical details of applying allometric scaling and maturation to PK parameters, the Bateman model and the Dost model. The results from the evaluation of the naïve designs are also displayed [28-31]. [file 1475-2875-10-181-S1.PDF]

Graphical display of the optimal designs

Figure S1 provides a graphical display of the optimal sampling times and windows for each design (see the Figures and Tables section at the end of this document).

Details of allometry and maturation on model parameters for the design with adults and children

For the optimal design with non-pregnant adults and children, oral clearance for an individual i (CL/F_i) and apparent volume of distribution (V/F_i) were defined as follows:

$$
\begin{aligned}
CL/F_i &= \left(\frac{CL/F_{pop}}{3.1}\right) \times \left(\frac{\text{weight}_i}{\text{weight}_{pop}}\right)^{0.75} \\
&\times \left[\exp(-k_{mat} \times \text{age}_i)\right] \\
&+ CL/F_{pop} \left(\frac{\text{weight}_i}{\text{weight}_{pop}}\right)^{0.75} \\
&\times \left[1 - \exp(-k_{mat} \times \text{age}_i)\right]
\end{aligned}
$$

$$
V/F_i = V/F_{pop} \times \left(\frac{\text{weight}_i}{\text{weight}_{pop}}\right)
$$

CL/F_{pop} and V/F_{pop} represent the typical oral clearance value and apparent volume of distribution value for the adult population, and weight_{pop} represents a typical weight for the adult population. CL/F for an infant was also allowed to mature to an allometrically scaled adult value according to age (months) to reach the population value CL/F_{pop}, hence k_{mat} is a first-order rate constant that determines the rate at which an infant's clearance approaches the allometrically scaled value of CL/F_{pop} [28]. This oral clearance maturation calculation assumes that the ontological maturation process reflects the same rate as that of the kidneys, however, DHA is cleared hepatically, with UDP-glucuronosyltransferase (UGT) 1A9 and 2B7 being the predominant isoforms involved in the clearance of DHA in humans [29]. Although DHA is not renally cleared, the expression above is a conservative assumption on the rate of maturation and others have made similar assumptions about hepatically cleared drugs [30].

In `POPT`, CL/F_{pop} and V/F_{pop} were set to the values determined in the analysis of the non-pregnant adults (Table 1) and the population weight (weight_{pop}) was set to the median weight of the non-pregnant adults, 51 kg. k_{mat} was set to 0.082, which assumes CL/F maturates at the same rate that the glomerular filtration rate (GFR) matures. The age/weight combinations that were used were based on the linear model mentioned in the methods section, and ranges of these combinations were considered for each age group. The resulting values for CL/F and V/F considered for the designs are displayed in Figure S2 (see the Figures and Tables section).

Mathematical details of the Bateman and Dost models

The Bateman model

Under the Bateman model, the DHA concentration C for an individual i at blood sampling event j is given by

$$
\begin{aligned}
C_{ij} &= \frac{D_i \times k_{a,i}}{V/F_i \times k_{a,i} - CL/F_i} \\
&\times \Bigg[\exp\left(-\frac{CL/F_i}{V/F_i} \times (t_{ij} - t_{lag,i})\right) \\
&- \exp(-k_{a,i} \times (t_{ij} - t_{lag,i}))\Bigg] + \varepsilon_{ij}, \\
\text{for} \quad & t_{ij} > t_{lag,i}.
\end{aligned}
$$

D_i and t_{ij} represent the dose administered to an individual and the time post-dosing of blood sampling event j, respectively. $t_{lag,i}$ represents an individual's lag-time, which is the time between drug administration and start of absorption. $k_{a,i}$, CL/F_i and V/F_i represent an individual's absorption rate constant (/h), oral clearance (L/h) and apparent volume of distribution (L), respectively (the PK parameters). The F in these expressions is the fraction of the dose absorbed. The individual PK parameters and lag-times were assumed to be random and log-normally distributed, that is,

$$\theta_i = \theta \exp(\eta_{\theta,i}),$$

where θ_i and θ represent the individual and population parameters, respectively, and $\eta_{\theta,i}$ is a subject-specific random-effect for θ assumed to follow a normal distribution with a mean of zero and variance

Ω_θ. Thus Ω_θ is the between-subject variance (BSV) for a parameter θ. The residual errors ε_{ij} were assumed to be normally distributed with a mean of zero and variance σ^2, thus σ^2 represents the residual variability. A proportional error structure was fitted, that is, the residuals were assumed to be proportional to the estimated individual drug concentrations. For the optimal designs, an additive component of the residual variance was also specified to ensure the sampling times would not occur at times where many of the samples would be below the lower limit of quantification.

The Dost model

The structural model proposed by Dost [31] is given by

$$\begin{aligned} C_{ij} &= \frac{D_i}{V/F_i} \times k_i \times (t_{ij} - t_{lag,i}) \\ &\times \exp(-k_i \times (t_{ij} - t_{lag,i})), \\ \text{for} \quad & t_{ij} > t_{lag,i}. \end{aligned}$$

As with the Bateman model, individual parameters were assumed to be random and log-normally distributed, residual errors were assumed to be proportional to the estimated individual drug concentrations and the model was fitted with and without a lag-time.

Comparison of the optimal designs with naïve designs

Based on constraints identified in the questionnaire and a visual inspection of the population PK profiles for the target populations, the naïve sampling times were decided to be at 0.5, 1 and 4 hours post-dosing for adults (both pregnant and non-pregnant), 0.5 and 2 hours for children older than two years and one hour for children less than two years.

Table S1 (see the Figures and Tables section) displays the empirical %RSEs of the Bateman model parameters assuming these naïve sampling times, as well as the empirical %RSEs for the optimal designs. For all patient groups, the %RSEs from the naïve designs were notably larger for k_a and V/F compared to the optimal designs. For the non-pregnant adult only and non-pregnant adult and children designs, the %RSE for the BSV of k_a was substantially larger for the naïve schedules. For the pregnant women, the empirical %RSE of the BSV of CL/F under the naïve design was twice that of the optimal design. Thus for the Bateman model, the optimal designs provided substantial improvement in parameter precision compared with the naïve designs.

Figures and tables

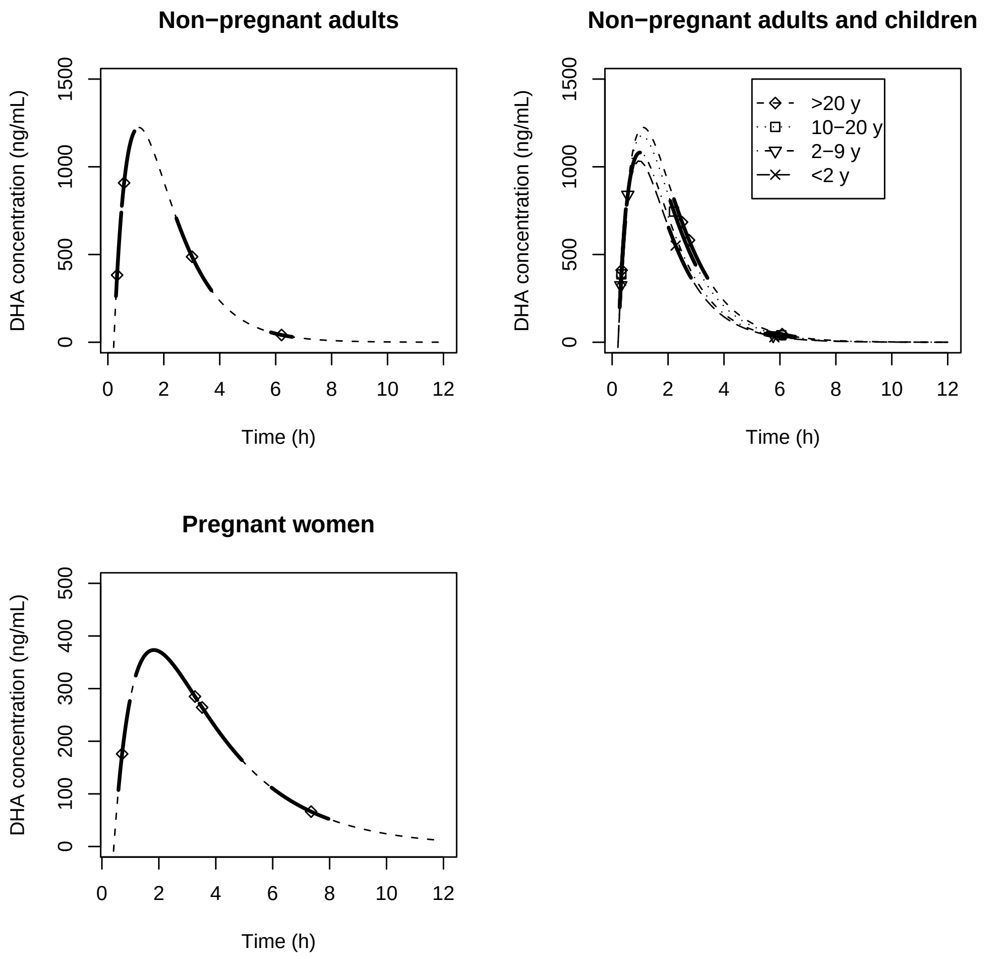

Figure S1: Optimal sampling times (open symbols) and windows (thick lines) superimposed on pharmacokinetic profiles for each target population.

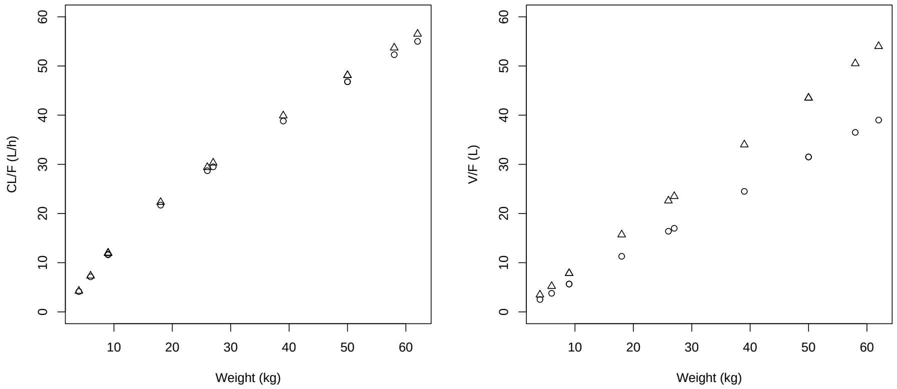

Figure S2: Values of CL/F and V/F used for the design with non-pregnant adults and children. Open circles represent the Bateman model with a lag-time and open triangles represent the Bateman model without a lag-time.

Table S1: Empirical percent relative standard errors (%RSEs) for the Bateman model assuming the naïve and optimal designs

	k_a	CL/F	V/F	t_{lag}	Ω_{k_a}	$\Omega_{CL/F}$	$\Omega_{V/F}$	σ
**Non-pregnant adults**								
Optimal design								
Simulation-estimation†,‡	10.7	5.99	17.3	6.86	52.9	30.7	21.6	7.87
Naïve design								
Simulation-estimation†,‡	331	6.78	44.8	21.7	4.5×10^9	42.3	28.2	10.1
**Non-pregnant adults and children**								
Optimal design								
Simulation-estimation†,‡	8.97	5.63	22.7	9.88	55.2	37.4	29.2	8.52
Naïve design								
Simulation-estimation†,‡	90.9	7.08	54.0	29.0	232	44.0	39.7	12.8
**Pregnant women**								
Optimal design								
Simulation-estimation$^{\dagger,\sharp}$	14.7	8.28	17.5	8.67	-	27.0	25.8	7.33
Naïve design								
Simulation-estimation$^{\dagger,\flat}$	46.1	16.5	32.8	2.74	-	60.3	24.8	8.84

†Empirical %RSEs

$^{\ddagger}\Omega_{t_{lag}}$ fixed

$^{\sharp}\Omega_{t_{lag}}$ and Ω_{k_a} fixed

$^{\flat}\Omega_{t_{lag}}$ omitted and Ω_{k_a} fixed
